# Modified platelet deposition on matrix metalloproteinase 13 digested collagen I

**DOI:** 10.1111/jth.13166

**Published:** 2015-11-30

**Authors:** J.‐M. Howes, N. Pugh, V. Knäuper, R. W. Farndale

**Affiliations:** ^1^ Department of BiochemistryUniversity of CambridgeCambridgeUK; ^2^ Department of Biomedical and Forensic ScienceAnglia Ruskin UniversityCambridgeUK; ^3^ Cardiff University Dental SchoolCardiffUK

**Keywords:** collagen, collagenase, platelets, GPVI collagen receptor, matrix metalloproteinase 13

## Abstract

**Background:**

Atherothrombosis underlies acute coronary syndromes, including unstable angina and acute myocardial infarction. Within the unstable plaque, monocytes express collagenolytic matrix metalloproteinases (MMPs), including MMP‐13, which degrades fibrous collagen. Following rupture, vessel wall components including degraded collagen are exposed to circulating platelets. Platelet receptors then mediate the recruitment and activation of platelets to form a thrombus, blocking blood flow and resulting in myocardial infarction and sudden death.

**Objectives:**

Here we aim to provide information on the effects of collagen degradation on platelet adhesion and thrombus formation.

**Methods:**

Using increasing concentrations of MMP‐13, we induced progressive degradation of fibrous and monomeric collagen I, visualized by electrophoresis, and then investigated the capacity of the resulting fragments to support static platelet adhesion and thrombus formation in whole flowing blood.

**Results:**

Both integrin and glycoprotein VI‐dependent interactions with fibrous collagen underpin high levels of platelet adhesion under both conditions, with little obvious effect of MMP‐13 treatment. Static platelet adhesion to monomeric collagen was strongly α2β1‐dependent regardless of degradation status. Under flow conditions, partially degraded monomeric collagen supported increased thrombus deposition at 10 μg mL ^−1^ MMP‐13, falling close to background when collagen degradation was complete (100 μg mL ^−1^ MMP‐13).

**Conclusions:**

New binding activities come into play after partial digestion of collagen monomers, and net platelet‐reactivity through all axes is abolished as degradation becomes more complete.

Following atherosclerotic plaque rupture, circulating platelets bind to exposed vessel wall collagens, where they initiate arterial thrombosis 1. Specific receptors mediate platelet recognition of and adhesion to collagen. GPIbα binds to immobilized von Willebrand factor (VWF) in the vessel wall, initiating platelet capture 2, and glycoprotein (GP)VI binds directly to collagen and activates platelets. Integrin α2β1 stabilizes the early stages of the platelet‐collagen interaction, and integrin αIIbβ3 supports platelet‐platelet interactions mediated by fibrinogen and VWF.

During plaque development, smooth muscle cells deposit excess collagens I and III within the subendothelial intimal space 3. Collagen is resistant to most proteolytic degradation, and whilst healthy arteries do not express active collagenases, in the unstable plaque macrophages secrete matrix metalloproteinase (MMP)‐1 and MMP‐13, which cleave collagen^4^, ^5^, ^6^, leaving behind bioactive fragments that may target cells downstream from the site of injury 3. Despite this, little is known about the effects of progressive collagen proteolysis on platelet recognition. Here we investigate the relationship between MMP‐13 treatment and platelet adhesion to fibrous and monomeric collagen I under static and shear conditions.

## Methods

### Proteolytic digestion of collagen I by MMP‐13

ProMMP‐13 was activated using 1 mm 4‐aminophenylmercuric acetate for 1 h at 37 °C, and dialysed at 4 °C against 50 mm TBS. Active MMP‐13 (0–100 μg mL^−1^) was added to either monomeric (Devro, Chryston, Scotland) or fibrous (Ethicon Corp., Somerville, NJ, USA) bovine collagen I (1 mg mL^−1^) and incubated for 16 h at 37 °C.

### SDS‐PAGE of collagen samples

Digested collagens were separated by reducing electrophoresis on 4–12% NuPage^®^ bis‐tris gels (Invitrogen, Paisley, UK) and Coomassie stained.

### Human washed platelet preparation

Venous platelets were isolated from healthy volunteers as previously described 7 and counted using a Z2 counter (Beckman Coulter, High Wycombe, UK).

### Adhesion assays

Platelet adhesion assays were conducted as previously described 7. The anti‐αIIbβ3 compound GR144053 (4‐[4‐[4‐(aminoiminomethyl]‐1‐piperazinyl]‐1‐piperidineacetic acid hydrochloride trihydrate) was purchased from Calbiochem, Watford, UK. The α2β1‐binding peptide, GFOGER (GCP(GPP)_5_‐GFOGER‐(GPP)_5_‐GCP) courtesy of Dr Dominique Bihan) and anti‐GPVI scFv were generated as previously described 8, 9. The anti‐α2β1 monoclonal antibody was a gift from Dr Barry Coller, Rockefeller University, New York. Ninety‐six‐well Immulon^®^ 2HB plates (Nunc, Thermo Scientific, Paisley, UK) were coated with 1 μg digested collagen/MMP‐13 solution or the controls (GFOGER or bovine serum albumin (BSA)) in 100 μL 0.01 m acetic acid, and left overnight at 4 °C. Washed platelets (100 μL at 1.5 × 10^8^ mL^−1^) in calcium free tyrodes (CFT) containing 2 mm Mg^2+^ or EDTA were allowed to adhere for 1 h following pre‐incubation for 20 min at room temperature with GR144053 (10 μm) or anti‐GPVI/α2β1 (10 μg mL^−1^) as indicated. The wells were washed, lysed and platelet binding quantified using a colorimetric alkaline phosphatase assay 7.

### Whole blood perfusion experiments

Blood was collected into 40 μm D‐Phenylalanyl‐L‐prolyl‐L‐arginine chloromethyl ketone (PPACK, Enzo Life Sciences, Exeter, UK), supplemented hourly with 10 μm PPACK, and mixed with 1 μm 3,3'dihexyloxacarbocyanine iodide dye (DIOC_6_) 15 min before use. Glass coverslips were coated with (10 μg) collagen solution in 0.01 m acetic acid and left overnight at 4 °C. Slides were blocked with bovine serum albumin (BSA) (1% w/v) for 30 min and blood then drawn through the chamber at a wall shear rate of 1000 s^−1^, mimicking arteriolar conditions. Images (field size 360 × 360 μm) were taken from three separate areas of the slide; 0.69‐μm Z–stacks encompassing the entire thrombus height were collected using an Olympus FV 300 confocal microscope and an UplanFLN 40x NA1.30 oil immersion objective and processed using ImageJ1.35 software (NIH, Bethesda, MD, USA) to determine thrombus volume 10.

## Results and discussion

### Visualization of collagen degradation by SDS‐PAGE

**Figure 1:**
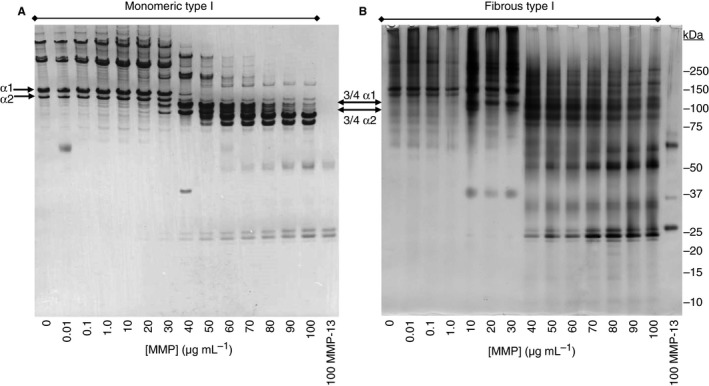
Visualization of (A) monomeric and (B) fibrous collagen I degradation by SDS‐PAGE under reducing conditions. Activated matrix metalloproteinase (MMP)‐13 (total volume of 10 μL) was added to the collagens and degradation allowed to proceed for 16 h at 37 °C. Gels are representative of nine repeated experiments (*n* = 9). Intact collagen I α1 and α2 chains and their ¾ fragments are marked for reference.

Proteolysis of monomeric collagen I is proportional to enzyme concentration, with the α1 and α2 chains disappearing at 40 μg mL^−1^ and any background smearing above 150 kDa clearing with progressive proteolysis. MMP‐13 acts at the collagenase cleavage site, located at Gly906‐Leu907, ¾ of the way along the collagen α chain 11. The ¾ fragments are visible at ~75 and 90 kDa, and the ¼ fragment at 25 kDa alongside an increasing level of autolysed MMP‐13 12 (Fig. 1A). For monomeric collagen, proteolysis at 37 °C yields an additional, stable band below the ¾ fragments not seen at 24 °C (data not shown). As expected, some of the large cross‐linked fibrous collagen is unable to enter the gel, and so degradation is observed first as increased density at 250–100 kDa in the 10–30 μg mL^−1^ lanes, then as a decrease in aggregate material above 150 kDa (Fig. 1B). Following incubation at 37 °C for 16 h, the MMP‐13 control has undergone virtually complete autolysis into small fragments 13.

The structure of a collagenase is highly conserved, comprising pro‐peptide, catalytic (Cat) and hemopexin (Hpx)‐like domains 14. Cat and Hpx cooperate to recognize and bind collagen adjacent to its cleavage site 15 with Hpx‐mediated helix relaxation enabling hydrolysis. Subsequent gelatinolytic degradation of the clipped α chains by MMP‐13 16, 17 has the potential to influence recognition of collagen by platelet adhesion receptors, and these data are presented below.

### Static platelet adhesion to MMP‐degraded collagens

**Figure 2:**
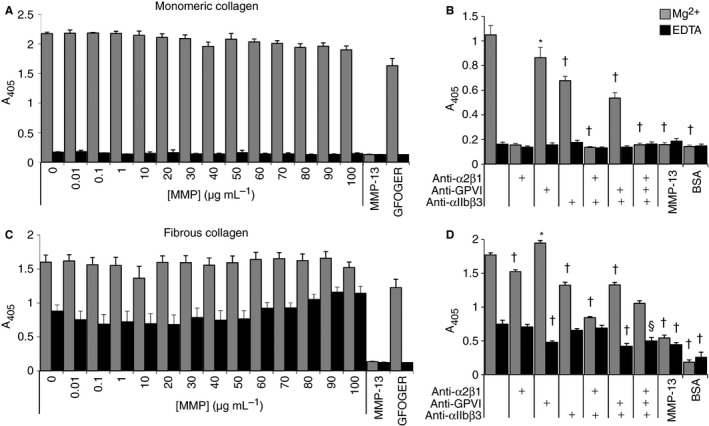
(A). Adhesion of washed platelets to ELISA wells coated with matrix metalloproteinase (MMP)‐13‐digested monomeric collagen, the α2β1 recognition peptide GFOGER and a negative control (bovine serum albumin, BSA). (B) Platelet adhesion to monomeric collagen I in the presence of anti‐α2β1 and anti‐GPVI antibodies or anti‐αIIbβ3 compound where stated. (C) Adhesion of washed platelets to ELISA wells coated with MMP‐13 digested fibrous collagen solution and the α2β1 collagen recognition peptide GFOGER. (D) Platelet adhesion to fibrous collagen I in the presence of anti‐α2β1, anti‐GPVI antibodies or anti‐αIIbβ3 compound where stated. Platelets were allowed to adhere for 1 h at room temperature prior to washing, lysis and assay as described in the Methods section. Experiments were performed in the presence of 2 mm Mg^2+^ (grey bars) or 2 mm EDTA (black bars). Data represent the mean ± SE of at least three experiments. In B and D, *P < 0.05, ^§^ *P* < 0.01, ^†^ *P* < 0.001 (one‐way anova and Dunnett's multiple comparison test) relative to untreated platelets in either the presence of Mg^2+^ or EDTA, as appropriate.

**Figure 3:**
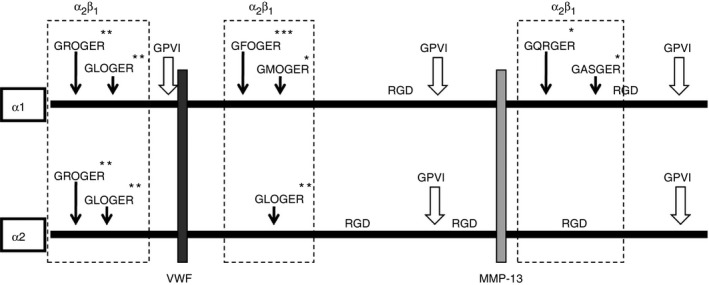
Schematic representation of key sites in the collagen I α1 and α2 chains. The matrix metalloproteinase (MMP)‐13 cleavage site, von Willebrand factor (VWF) binding site and GPVI binding sites are denoted. Also represented are: *low‐, **intermediate‐ and ***high‐affinity GxxGER α2β1 integrin recognition sites.

Mg^2+^‐dependent, integrin‐mediated static platelet adhesion to monomeric collagen I 7, 8 remained unchanged regardless of the extent of collagen degradation (Fig. 2A). Pre‐incubation with EDTA or an anti‐α2β1 antibody (subsequent, separate experiments) abolished all adhesion, confirming that the interaction absolutely requires integrin α2β1, with only modest contribution from GPVI (Fig. 2B). Messent *et al*. 12 found that the linear ¼ fragment did not bind α2β1. Together, these data suggest that platelet adhesion to MMP‐13‐digested monomeric collagen is primarily mediated through the ¾ fragment (where GFOGER and other high‐affinity α2β1‐binding sites reside 18; see Fig. 3). In this scenario, the MMP‐13‐mediated loss of the weak α2β1‐binding sites and the C‐terminal Gly‐pro‐Hyp‐rich tract, a putative GPVI recognition motif present in the ¼ fragment, make little discernible contribution to static platelet binding. Autolysed MMP‐13 did not support platelet adhesion.

Static platelet adhesion to fibrous collagen I displayed a constant Mg^2+^‐dependent element regardless of MMP‐13 treatment (Fig. 2C), which was reduced but not abolished by EDTA, suggesting both integrin‐dependent and integrin‐independent contributions. The anti‐GPVI scFv in the presence of EDTA reduced platelet adhesion by ~70% relative to control and by ~40% relative to EDTA alone (each *P* < 0.001, one‐way anova and Dunnett's test), showing that GPVI contributes to integrin‐independent binding. Although α2β1 and/or αIIbβ3 inhibition also reduced integrin‐dependent adhesion (*P* < 0.001), neither blockade affected metal ion‐independent binding (Fig. 2D).

In order to bind to collagen fibers, GPVI dimerises on the platelet surface 19. Our results suggest that whilst fibrous collagen provides ample accessible sites, the platelet GPVI dimer has little affinity for collagen monomers or proteolytic fragments and so is unlikely to support platelet adhesion to digested collagen. Some adhesion to fibers remains following combined α2β1, GPVI and αIIbβ3 blockade, suggesting contributions by other mechanisms. Further studies may identify the receptors responsible.

### Flow‐dependent thrombus formation of whole blood is differentially regulated on digested monomeric collagen

Shear stress is critical for VWF‐dependent platelet recruitment to collagen. In flowing blood, platelet attachment to collagen is initiated by GPIbα binding to the A1 domain of immobilized VWF, the first step in thrombus formation 20, 21. Using specific collagen‐derived peptides, we observed that VWF is required for thrombus deposition at high shear rates, together with either high‐affinity α2β1 or GPVI, or preferably both 10.

**Figure 4:**
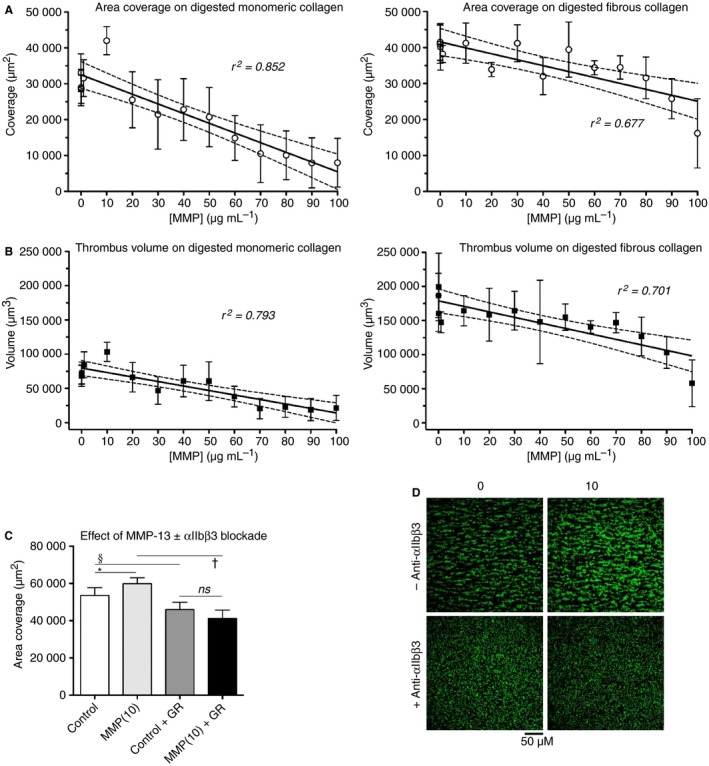
Deposition of platelets on matrix metalloproteinase (MMP)‐13 digested monomeric or fibrous collagen I in whole blood under flow conditions. Surfaces were prepared and blood was drawn through the perfusion chamber for 5 min as described in the Methods section. Results shown are mean values ± SEM plotted vs. [MMP‐13] used to digest the collagen. 95% confidence intervals are also shown for the best fit lines, calculated by linear regression (Prism 5 for Mac OSX). (A) Surface coverage of platelets on monomeric collagen (left) or fibrous collagen (right), *n* ≥ 4, combining all data from nine experiments. (B) Thrombus volume measured in the same experiments. (C) The mean surface coverage from additional experiments using monomeric collagen with or without treatment with 10 μg/mL^−1^ MMP‐13, performed in the presence or absence of the αIIbβ3 antagonist, GR144053 (*n* = 7); *P < 0.05, ^§^ *P* < 0.01, ^†^ *P* < 0.001. (D) Representative images of platelet aggregates from (C).

The first experiments using digested monomeric collagen showed a linear decline, *P* < 0.0001, in surface coverage with MMP‐13 level, from the control value of 33 000 μm^2^ to 8000 μm^2^ after treatment with MMP‐13 at 100 μg mL^−1^ (Fig. 4A, left panel). One point (10 μg mL^−1^) was markedly above the 95% confidence range, at a value of 42 000 μm^2^. A corresponding fall in thrombus volume, *P* < 0.0001, (Fig. 4B, left panel) was observed, again with an outlying value at an MMP‐13 level of 10 μg mL^−1^. This modest rise in platelet adhesion suggests increased accessibility of cryptic motifs (such as Arg‐Gly‐Asp (RGD); inactive in triple‐helical collagens, but active in linear peptides), which may engage integrins other than α2β1 such as αIIbβ3. To examine this effect of MMP‐13 at 10 μg mL^−1^ more closely, seven further experiments were conducted with or without pre‐incubation of whole blood with the αIIbβ3 antagonist, GR144053. These experiments showed significantly greater surface coverage (*P* < 0.05, repeated measures anova) after MMP‐13 treatment, from 53 500 to 60 000 μm^2^, confirming the anomalous point in Fig. 4(A,B). GR144053 reduced mean surface coverage from 47 500 and 55 500 μm^2^ on control and MMP‐13‐treated collagen monomers (10 μg mL^−1^) to 46 000 and 41 000 μm^2^, respectively (*P* < 0.01 and 0.001, respectively, Fig. 4C). The antagonist reduced the thrombi to a lawn of single platelets (Fig. 4D). Importantly, the elevated surface coverage after MMP‐13 treatment was abolished by αIIbβ3 blockade. These data are consistent with an increase in either the αIIbβ3‐reactivity of collagen monomers after limited digestion or their ability to activate αIIbβ3; the former explanation appears more likely; we know of no activatory receptor that is regulated by non‐helical collagens. It is worth noting that inter‐donor differences accounted for over half the variance observed here.

On MMP‐13‐treated fibrous collagen, platelet surface coverage and volume followed a similar pattern, falling in a linear fashion towards zero (*P* = 0.0003 and 0.0002, respectively) (Figs 4A,B, right panels). The data for both forms of collagen contrast with the static adhesion results, which showed no significant change in platelet adhesion regardless of the degree of collagen proteolysis. The thrombus volume reflects the aggregation of activated platelets, and is most likely higher for fibrous collagen than monomeric collagen as a result of the engagement of GPVI dimers by the former but not the latter. As the proteolysis of each collagen form continues, it is likely that these binding sites are all progressively destroyed, inevitably resulting in near‐total loss of platelet adhesion.

Although collagenolytic MMPs can cleave the collagen molecule at several loci 22, substrate specificity and thus proteolysis are strictly governed by the tightly regulated structure of the collagen fibril, resulting in preferential cleavage at the canonical ¾–¼ site. In the intact fibril, access to this site may be restricted by a C‐telopeptide, which must be removed to permit collagenolysis 23. However, the Cat domain of MMP‐13 can cleave the non‐helical N‐telopeptide of collagen I 24, and similar activity has been proposed at the C‐terminus. As degradation of the fibril proceeds, therefore, removal of the outer fibril components may reveal previously hidden cleavage as well as cell adhesion sites 23, 25. For fibrous collagen, our static adhesion experiments report that the sites available for platelet adhesion are both integrin‐ and GPVI‐dependent, which are abolished by the chelation of Mg^2+^ and the presence of an anti‐GPVI scFv, respectively (Fig. 2D).

In conclusion, proteolytic degradation of an intact collagen fibril within an atherosclerotic plaque is likely to be a complex and iterative process, determined by the accessibility of its collagenase cleavage sites. Platelet reactivity will vary as proteolysis proceeds, as buried or cryptic receptor binding sites within the fibril are revealed and as monomers or smaller fragments released. Ultimately, it seems likely that collagenase activity will reduce the fibril to small components that are unreactive under shear conditions and become dispersed.

## Addendum

V. Knäuper provided essential materials. N. Pugh assisted with flow experiments. R. Farndale designed the research, analysed the data and helped to write the manuscript and J. M. Howes designed and performed the research and wrote the manuscript.

## Disclosure of Conflict of Interests

The authors state that they have no conflict of interest.
